# Structure-Activity Relationship in TLR4 Mutations: Atomistic Molecular Dynamics Simulations and Residue Interaction Network Analysis

**DOI:** 10.1038/srep43807

**Published:** 2017-03-08

**Authors:** Muhammad Ayaz Anwar, Sangdun Choi

**Affiliations:** 1 Department of Molecular Science and Technology, Ajou University, Suwon 443-749, Korea

## Abstract

Toll-like receptor 4 (TLR4), a vital innate immune receptor present on cell surfaces, initiates a signaling cascade during danger and bacterial intrusion. TLR4 needs to form a stable hexamer complex, which is necessary to dimerize the cytoplasmic domain. However, D299G and T399I polymorphism may abrogate the stability of the complex, leading to compromised TLR4 signaling. Crystallography provides valuable insights into the structural aspects of the TLR4 ectodomain; however, the dynamic behavior of polymorphic TLR4 is still unclear. Here, we employed molecular dynamics simulations (MDS), as well as principal component and residue network analyses, to decipher the structural aspects and signaling propagation associated with mutations in TLR4. The mutated complexes were less cohesive, displayed local and global variation in the secondary structure, and anomalous decay in rotational correlation function. Principal component analysis indicated that the mutated complexes also exhibited distinct low-frequency motions, which may be correlated to the differential behaviors of these TLR4 variants. Moreover, residue interaction networks (RIN) revealed that the mutated TLR4/myeloid differentiation factor (MD) 2 complex may perpetuate abnormal signaling pathways. Cumulatively, the MDS and RIN analyses elucidated the mutant-specific conformational alterations, which may help in deciphering the mechanism of loss-of-function mutations.

Toll-like receptor 4 (TLR4), a type 1 transmembrane receptor, is vital for the innate immune response. When TLR4 senses lipopolysaccharides (LPS), it initiates a cascade of regulated immune responses that ultimately curb the bacterial challenge. TLR4 is composed of three domains: an ectodomain that contains 22 leucine-rich repeats (LRRs) and mediates LPS recognition and receptor dimerization; a transmembrane domain; and the Toll/interleukin-1 receptor domain (TIR domain), which is essential for downstream signal transduction^1^. However, TLR4 alone is not sufficient for the recognition of LPS; in conjunction with myeloid differentiation factor 2 (MD2), TLR4 forms a complex that is crucial for LPS recognition and antibacterial immune responses. MD2 is associated with the extracellular domain of TLR4, which directly binds LPS and confers LPS responsiveness to TLR4^2^. TLR4 can signal via the myeloid differentiation primary response gene 88 (MyD88) and TIR domain-containing adaptor inducing interferon (TRIF) pathways^3^; excessive TLR4 signaling in response to LPS often causes fatal septic shock because of uncontrolled inflammation^4^.

TLR4 is highly polymorphic as revealed by sequence analyses conducted in various species, however the structure is conserved^5^. The extracellular region of TLR4 harbors many sequence variations; in particular, the mutations D299G and T399I abrogate responsiveness to LPS^6^. These mutations are widely studied with respect to their role in pathogenic and non-pathogenic diseases. These loss-of-function mutations cause decreased airway responsiveness after stimulation with LPS^6^ and are implicated in multiple diseases caused by gram-negative bacteria^6^,^7^ or the respiratory syncytial virus^8^. Single nucleotide polymorphisms (SNPs) of TLR4 are linked to a reduced risk of noninfectious diseases, such as atherosclerosis^9^, rheumatoid arthritis^10^, diabetes mellitus type 2^11^ and Alzheimer’s disease^12^. However, it is likely that the impact of the D299G polymorphism on sepsis is restricted to gram-negative infections because it does not affect polymicrobial sepsis^13^.

The underlying mechanism of abrogated TLR4 function caused by these mutations is unclear. Mutated TLR4 shows reduced expression on the cell surface in embryonic kidney cells and human airway epithelial cells^6^,^14^; however, this observation is challenged by the results of another study^15^. Furthermore, experiments using equal concentrations of wild-type and mutated forms of TLR4 in HEK293T cells have shown that neither TLR4^G299^ (TLR4 D → G at position 299) nor TLR4^I399^ (T → I) can activate NF-κB. Additionally, cytokine gene expression responds poorly to the respiratory syncytial virus when cells are treated with LPS, chlamydial heat shock protein 60, and F protein^15^,^16^. The D299G/T399I TLR4 mutant has high affinity for LPS compared with that of wild-type TLR4 (TLR4^WT^); however, the D299G/T399I is more dependent on MD2 for proper cell-surface expression^19^. The D299G mutation has been shown to alter TLR4 signaling by interfering with the recruitment of MyD88 and TRIF^17^. However, a recent study contradicts this result, showing that mutant forms may trigger a higher expression of interferon (IFN)-β mRNA than that triggered by TLR4^WT18^. These results indicate that TLR mutations in humans dictate the extent of the innate immune response in cellular anomalies and may shape the progression of other human pathologies.

Crystallography has shed light on ligand binding and subsequent dimerization of TLR4/MD2^19^,^20^. These crystal structures show the dimer arrangement, spatial orientation of the individual chains, and changes in complex formation induced by the binding of ligands. Furthermore, numerous computational studies have evaluated the various phenomena related to TLR4 signaling^21^,^22^,^23^,^24^,^25^,^26^. The recently revealed crystal structure of polymorphic TLR4 has provided insight into how these mutations affect signaling^20^. Intriguingly, T399I has no effect on the structure of wild type and mutant TLR4, whereas D299G may locally disrupt the structure. The D299G substitution influences the three-dimensional structure because a charged amino acid is replaced with a neutral one. However, from a structural point of view, TLR4 is tolerant of this mutation; the overall structure remains intact and binds successfully to MD2 and LPS. Thus, the local conformational change does not severely hamper the homo- and heterodimerization of TLR4.

Computational approaches and molecular dynamics simulations (MDS) are frequently used to decipher mutation-induced changes in protein structure and function^27^,^28^. We performed MDS to evaluate localized effects in a dynamic mode and determined that in addition to the structural variation observed in the crystal structure, altered secondary structure, differential dominant motions, and anomalous residue signaling pathway may contribute to the abrogated function of TLR4. This study will fundamentally advance the mechanism of malfunctioning of TLR4/MD2 complex, and it can formulate the guidelines for TLR4 dependent treatment of various diseases.

## Results

### Structural features of TLR4

The structural features of TLR4 were elucidated via crystallography, which provides a plethora of information regarding the loss-of-function mutations associated with conserved structural features. However, protein function mostly depends on the dynamic nature of the protein. The static pose of the protein, estimated by crystallography, is useful for understanding loss of function, but does not provide details on the properties of the dynamic system. To bridge this gap, we performed extensive MD simulations (~1.8 μs in total) to investigate the dynamic function with respect to loss-of-function mutations, such as T399I and D299G, in TLR4. The average number of water molecules around the complexes in the first hydration shell (<0.4 nm) as computed by built-in GROMACS tool (gmx trjorder) was comparable (TLR4^WT^ = 5373.13 ± 34.12; TLR4^GI^ = 5355.35 ± 31.44; TLR4^I399^ = 5374.04 ± 36.01); however, in TLR4^G299^, the number was slightly higher (5439.7 ± 32.37).

**Figure 1:**
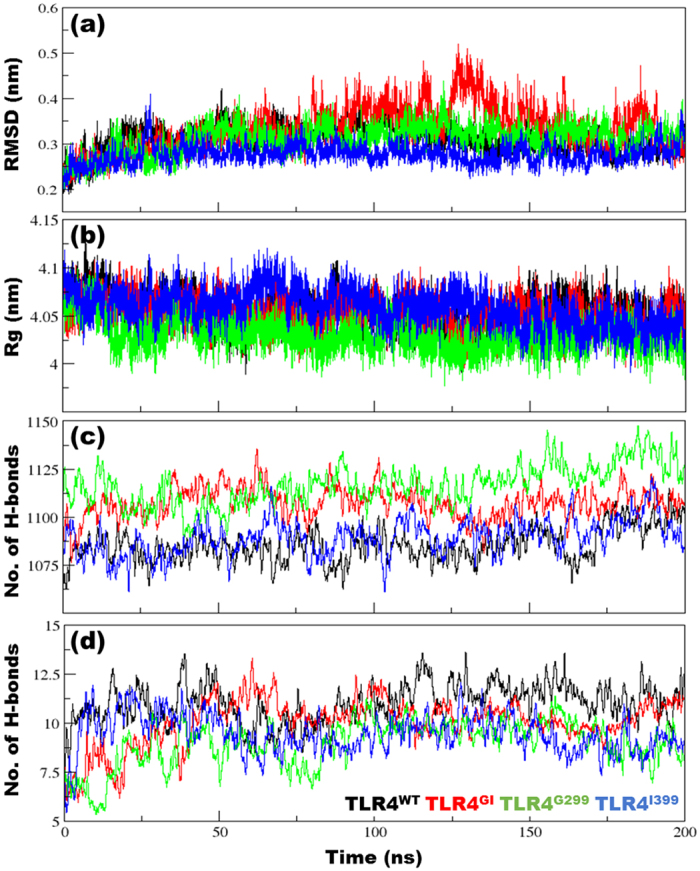
Structural parameters of Toll-like receptor (TLR4) variants. (**a**) Root mean-square deviation (RMSD) of backbone atoms after a least-squares fit to the initial structure over the whole trajectory. (**b**) Radius of gyration (Rg) of the backbone atoms in all complexes over the entire duration of simulation. (**c**) Number of intra-protein hydrogen bonds (H-bonds) and (**d**) protein-lipopolysaccharide (LPS) H-bonds for the entire duration of simulation. The criteria to measure H-bonds are based on cutoffs for the hydrogen-donor-acceptor angle (30°) and the donor-acceptor distance (0.35 nm). The OH and NH groups were regarded as donors, whereas O and N were acceptors.

The root mean-square deviation (RMSD) analysis measures the average distance between the selected atoms of superimposed biomolecules, indicating the closeness between three-dimensional structures. The radius of gyration (Rg) denotes the mass-weighted RMS distance of a group of atoms from their common center of mass, indicating the global dimension of the protein. The RMSD and Rg analyses indicated that all four complexes were stable over time (Fig. 1a). The RMSD fluctuation was maximal for TLR4^GI^ and high for TLR4^WT^; however, Rg values were constrained, ranging between 4.0–4.1 nm for all variants (Fig. 1b). The number of intra-protein hydrogen bonds (H-bonds) in TLR4^WT^ and TLR^I399^ was consistently low, whereas in TLR4^GI^ and TLR4^G299^ it was high (Fig. 1c,d). In TLR4^G299^, the H-bonds are present uniformly throughout protein unlike other complexes, which might be the reason that this complex showed highest number of H-bonds (Supplementary Figure S1). In the protein-LPS H-bonding network, this number was the highest in the TLR4^WT^ complex compared with those in others.

**Figure 2:**
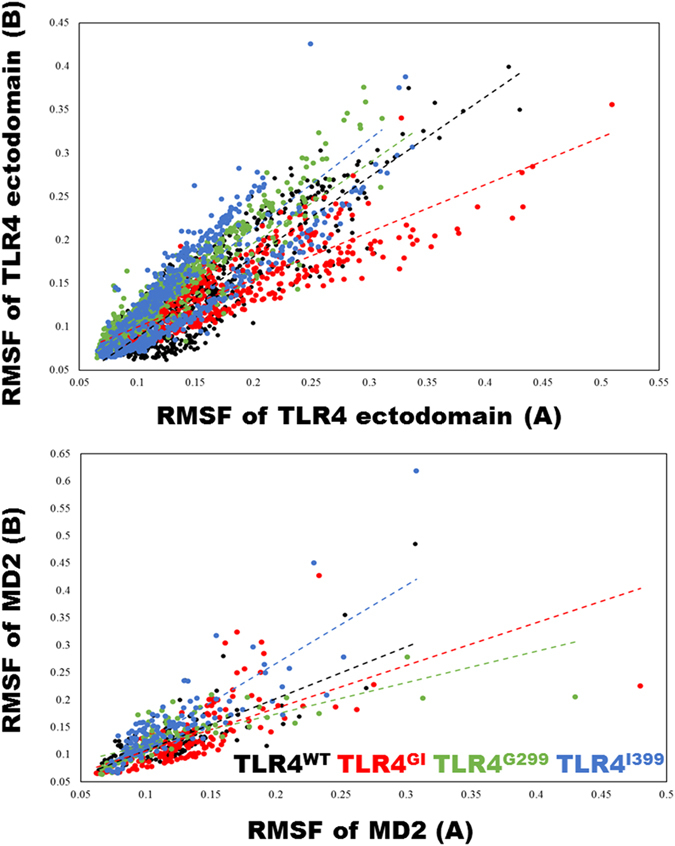
Root mean-square fluctuations (RMSF). RMSF of Cα from the last 100 ns of simulation. To elucidate the cohesiveness within the complex, the RMSF of chain A was compared against the RMSF of chain B in TLR4 and myeloid differentiation factor 2 (MD2). A linear trend line is drawn to highlight the divergence in complexes.

Amino acid residue fluctuation is an important measure in MDS because it can show how different residues in complexes behave on average. To evaluate internal fluctuation in various complexes, the root mean-square fluctuation (RMSF) of one chain was compared with that of the counter chain within the same complex. Because the number and nature of residues were identical, RMSF shows how protein dynamics are perturbed in complexes (Fig. 2). TLR4^WT^ showed a more linear RMSF spread than that of TLR4^GI^; however, the correlations between these RMSF plots were similar (TLR4^WT^ = 0.869; TLR4^GI^ = 0.87). TLR4^G299^ exhibited a spread that was more linear and correlated (0.944), while TLR4^I399^ showed a linear but less correlated RMSF (0.843); however, the magnitudes of spread were less than that of the corresponding TLR4^WT^ (s^2^ values are 0.00332, 0.0030, 0.00284 and 0.002796 for TLR4^WT^, TLR4^GI^, TLR4^G299^ and TLR4^I399^ respectively). Similarly, the RMSF of MD2 was mostly linear; however, the magnitude of the TLR4^WT^ complex was reduced. The correlation values were moderate for TLR4^WT^ (0.751), lowest for TLR^GI^ (0.653), and highest for TLR4^I399^ (0.838) and the values of variation in data (s^2^) are 0.00166, 0.00226, 0.00147, and 0.00251 for TLR4^WT^, TLR4^GI^, TLR4^G299^ and TLR4^I399^ respectively. Because the structure remained stable throughout the simulations, we determined the respective solvent exposure of protein and LPS. TLR4^WT^ and TLR4^GI^ had overlapping plots with minor differences. TLR4^WT^ showed no correlation in solvent-accessible surface area (SASA) values between the protein and LPS, while TLR4^GI^ was weakly positively correlated (Supplementary Figure S2). Although SASA distribution is distinctly different for TLR4^G299^ and TLR^I399^, however no correlation was observed.

The average distance between the chains was measured using the distance between two residues 299 and two residues 399 in their respective chains. TLR4^GI^ had a maximum distance at both positions (3.7 ± 0.11 and 5.18 ± 0.06 nm for residues 299 and 399, respectively, from one ectodomain to the other), while other complexes showed a comparatively reduced, but similar, range of distances [TLR4^WT^ (2.94 ± 0.17 nm; 5.07 ± 0.05 nm), TLR4^G299^ (2.84 ± 0.1 nm; 5.09 ± 0.04 nm), and TLR4^I399^ (2.79 ± 0.16 nm; 5.11 ± 0.05 nm)] (Supplementary Figure S3).

### Secondary structure alterations are prominent among different variants of TLR4

**Figure 3:**
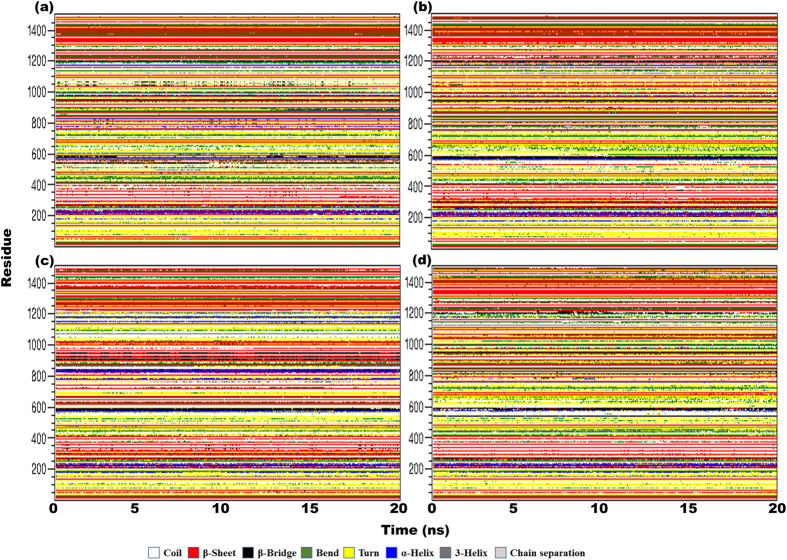
Secondary structure assessment over time. Secondary structures were calculated per the definition in the database of secondary structure assignments (DSSP) for the entire complex for the last 20 ns of each trajectory; structural evolution is represented in (**a**) TLR4^WT^, (**b**) TLR4^GI^, (**c**) TLR4^G299^, and (**d**) TLR4^I399^. Time is indicated on the *x*-axis, while residue numbers are indicated on the *y*-axis. The key describing the structural assignments is also shown.

Different variants of TLR4 are stable; therefore, we measured the evolution of secondary structure over time. The secondary structures of different regions were less stable than that of the overall structure (Fig. 3). Prominent variations were observed, and different regions acquired different secondary structure definitions over time. The highly variable parts included the region around position 299, which was either an α-helix or a β-turn, and the region around position 330, which exhibited a transition between a β-sheet, a 3_10_-helix, or other variants of secondary structure. Amino acids around 390 displayed variations in all conformations but primarily included β-sheets. The region around position 299, which comes in contact with MD2, displayed higher variation in secondary structure that may affect the spatial conformation of the complex. Variations in MD2 were also evident in different complexes, indicating that TLR4 exerts allosteric influence over the structure of MD2.

The propensity to form secondary structures also varied over time, particularly, when a single ectodomain was simulated (Supplementary Figures S4, S5). In TLR4^WT^, the propensity to form a coil structure increased over time, while the overall structure (including α-helixes, β-sheets, β-bridges, and β-turns) was slightly reduced. In TLR4^G299^, a higher number of amino acids adopted a structured format, and the propensity to form a coil was slightly decreased. The most prominent alteration was observed in the number of amino acids acquiring β-sheet conformation. Intriguingly, the propensity to form a π-helix was only observed in TLR4^G299^ but not in any other TLR4 variant. In TLR4^GI^ and TLR4^I399^, we observed stable structural features with minor variations in turn or loop regions; this is expected, given the structure of the TLR4 ectodomain. In simulations of the TLR4/MD2 complex, the structural features were mostly similar, likely because of the higher number of residues that masked the minor variations in the complex.

### Rotational correlation decay and density distributions are central to loss of function

**Figure 4:**
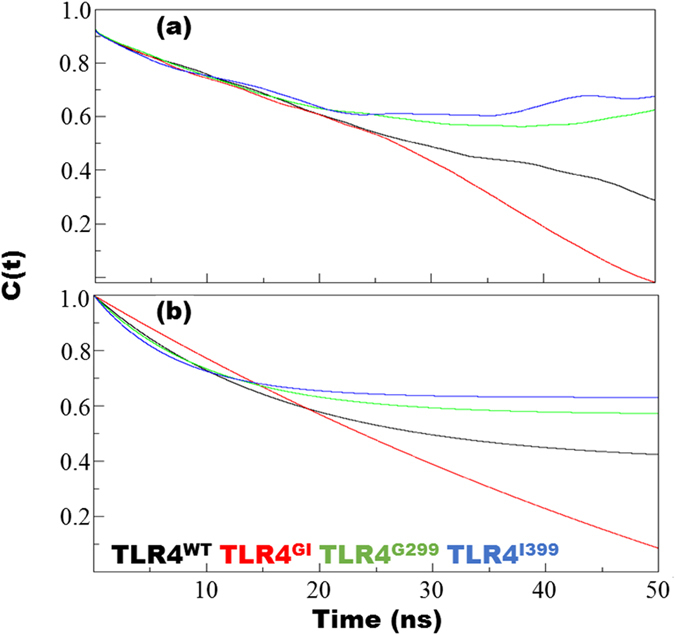
Rotational correlation function (RCF) of the NH bond vector of TLR4/MD2. The RCF was calculated for the last 100 ns from each trajectory using the second order Legendre polynomial (P2) of the NH bond vector (**a**) and a nonlinear curve fitted with the two parameters in a model-free approach (**b**). Fitting was performed according to the following formula: *y* = *a*0 + (1 − *a*0)*exp(−*x*/*a*1).

The evolution of the second-rank rotational correlation functions (RCF) of the NH bond vector for different variants of TLR4 were calculated according to the Legendre polynomial (P)2 and fitted to a second order parameter in a model-free approach (Fig. 4a). Consistent with previous observations^29^,^30^, all variants of TLR4 showed gradual decreases in the RCF during the first half, which can be attributed to fast internal motion, however, in second half, TLR4^GI^ showed a sudden decrease in RCF, while the rest of the variants showed a stabilized RCF. The linear decrease in TLR4^GI^ is unique because other systems show a logarithmic decrease in their RCF values. Linear decrease implies a uniform decay in fast internal motions; however, in other systems, exponential decrease implies a rapid decay in internal motions and a slower decrease in the more protracted motions of the complex. In this scenario, TLR4^WT^ showed a more pronounced pattern of decay compared with those of TLR4^G299^ and TLR4^I399^, which showed a stable plateau for the NH bond vector (Fig. 4b) and the values of the plateau were higher for TLR4^G299^ and TLR4^I399^. The rotational correlation time (τc) was at an intermediate level for TLR4^WT^ (28614.6 ± 4669.3 ps) (Supplementary Table S1). Surprisingly, τc was lower for TLR^GI^ and higher for variants with a single mutation. In comparison with single chain complexes, the τc values for hexameric complexes were expected to be twice as high; however, only a slight increase was observed for TLR^WT^. Similar behavior was observed with respect to the rotational diffusion constant (D_iso_).

### Flexibility of Cα atoms and conformational plasticity

**Figure 5:**
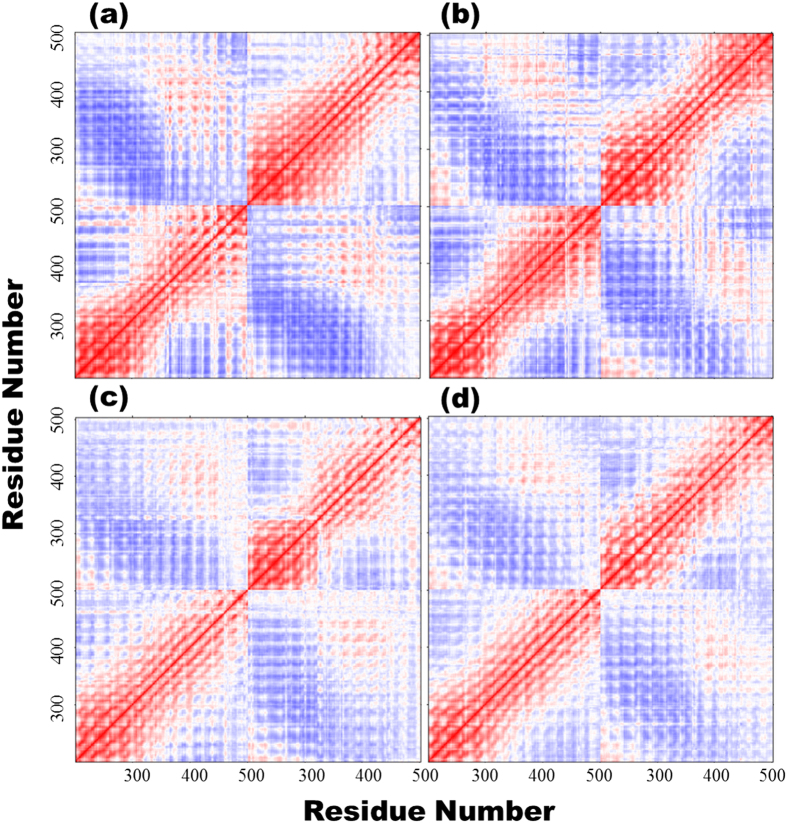
Correlation matrices for different TLR4 variants. The correlation of Cα atoms in the ectodomain of TLR4 from different variants is provided in matrix form in (**a**) TLR4^WT^, (**b**) TLR4^GI^, (**c**) TLR4^G299^, and (**d**) TLR4^I399^ with x- and y-axis denoting the atomic positions. In this figure, only residues from 200–500, which encompass the mutated regions, are included. For the full-length matrix, please see the Supplementary Figure S6.

An inter-residue correlation analysis was performed for Cα atoms to determine to which extent the movements were correlated among the atoms (Fig. 5 and Supplementary Figure S6). Correlative movements were similar among the variants; however, specific positive (red), and negative correlations (blue) were also evident. In the figure, the red diagonal line shows strong positive correlations and is indicative of autocorrelation of a residue. Moreover, the propensity for correlated and anticorrelated movements was higher in TLR4^WT^ than in the mutated ectodomains. Generally, TLR4^WT^ showed distinct pattern of correlative movements, for instance, the region around 350–450 highly correlative in both ectodomains of TLR4^WT^, whereas this region was less or uncorrelated in other complexes, and these movements were highlighted only when the central region (200–500 amino acids) was examined (Fig. 5). Therefore, the D299G and T399I mutations have a correlative effect on the cumulative movement displayed by these ectodomains.

**Figure 6:**
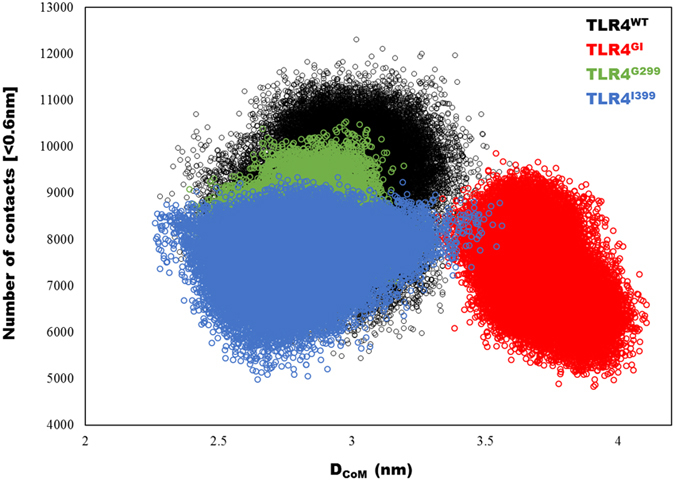
Contact intensity and TLR4/MD2 distance. The number of contacts between the dimers of TLR4/MD2, as well the distance between the center of mass of residue 299 from each domain, are plotted. Greater distance between the ectodomains of TLR4 and twisted geometry allow for a decreased number of contacts between the dimers.

The number of contacts between Cα atoms and any other atom within 6 Å have been calculated; and found that the contact intensity converged at a very close value (~8000) during the last 100 ns of the simulations (Supplementary Figure S7). To highlight the differences at the gross level, we compared the center of mass (COM) distance between residue 299 and contact intensity between the TLR4/MD2 dimers (Fig. 6). TLR4^WT^ had a higher number of contacts and lower distance between the residues, indicating that the wild type complex is more compact. Conversely, TLR^GI^ showed a lower number of contacts and higher inter-residue distance. TLR4^G299^ and TLR^I399^ exhibited lower inter-residue distance and lower inter-dimer contact intensity, indicating poor dimerization ability that might lead to inefficient signaling.

The relative position of the F126 side chain determines the agonistic and antagonistic behavior of TLR4^19^. Therefore, to predict the nature of complex, we measured the dihedral distribution of the F126 side chain on MD2. The distribution of χ1 (N-CA-CB-CG) and χ2 (CA-CB-CG-CD1) are shown in Supplementary Figure S8. In TLR4^WT^, χ1 was distributed at approximately −65°, whereas in TLR4^GI^, it was at approximately −84°. The distribution in the other two complexes fell in-between these values. TLR4^WT^, TLR4^GI^, and TLRR^G299^ displayed two clusters of χ2 at −100° and +85° with disparity in relative distribution. TLR4^WT^ showed an approximately equal distribution at both positions that implied a continuous flipping of bulky hydrophobic group of F126 residue; however, for other complexes, a higher distribution at the first position results in a lower distribution at the second position. Only TLR4^I399^ was found in its entirety at approximately +85°.

### Principal component analysis

Mutated TLR4 can form stable complexes (TLR4/MD2-LPS) but is unable to initiate the signal^20^. In order to identify dominant motions in complexes of TLR4, we performed principal components analysis (PCA), in which the first few eigenvectors capture the combined dominant motions whose amplitude quickly decreases to attain constrained and more localized fluctuations. PCA indicated that the first three PCs accounted for 59.9%, 52.3%, 43.5%, and 37.1% of the variance in the motion observed in the trajectories of TLR4^WT^, TLR4^GI^, TLR4^G299^, and TLR4^I399^, respectively. The magnitude of PC1 was the highest in TLR4^WT^; however, it was decreased by either one or two mutations.

**Figure 7:**
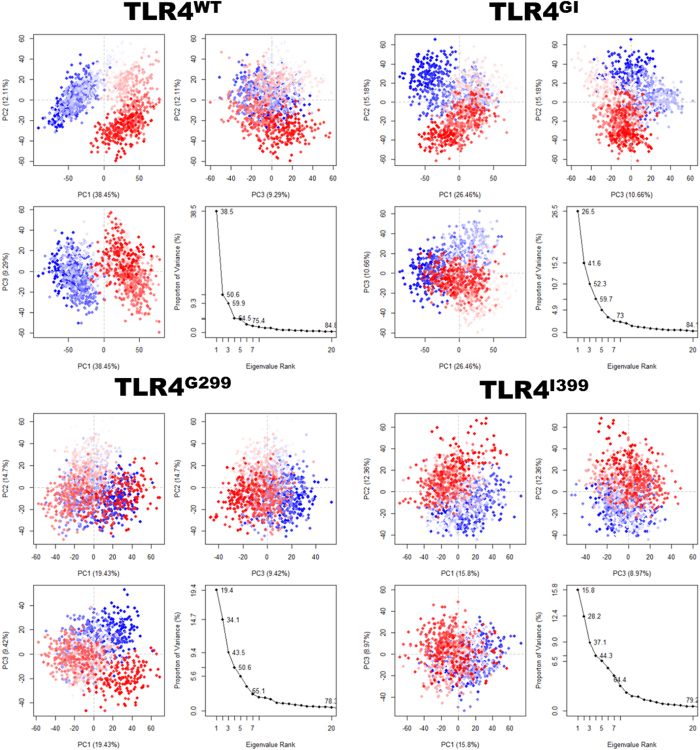
Principal component analysis (PCA). For the PCA, 1200 equidistance conformations were analyzed for the last 100 ns of each trajectory using the Bio3D package implemented in R. The first three eigenvectors, based on complex dominant motion, are extracted and compared. The variance captured by eigenvectors is also shown.

The two-dimensional plots between eigenvectors (EV) 1, 2, and 3 were drawn to compare the plausible conjoined movements (Fig. 7). The graph indicates the variance in the conformational distribution, where each dot represents one conformation of the complex. The continuous color representation (from blue to white to red) highlights the periodic jumps between these conformations. For TLR4^WT^, the subspace of PC 1/2 and 1/3 clearly showed the thermodynamically distinct periodic jumps with a substantial energy barrier. However, TLR4^GI^, TLR^G299^, and TLR4^I399^ showed a uniform and overlapping PC subspace that lacked an energy barrier. This analysis suggests that TLR4 may undergo a periodic shift in its conformation to reorient its TIR domain. Distinct clustering may be energetically costly; however, it may also provide a control mechanism. In mutated complexes, this periodic conformational shifting may be lost because of the perturbed internal motion caused by D → G and T → I mutations; these mutations may eliminate the critical correlated and coordinated motions in the ectodomains, which ultimately leads to confinement in one energy basin and loss of function.

The overlap between covariance matrices was calculated to examine the prominent directions of the TLR4/MD2 complexes. In comparing orthogonal matrices, the extent of the overlap is indicated by the normalized values of 1 for perfect overlap and 0 for no overlap and matrices are orthogonal. For this comparison, we used first 10 eigenvectors that accounted for >70% of the magnitude of the overall variance, and quantified the modes similarity that captured the largest deformational propensity in each structure (Supplementary Figure S9). All the variants were compared against TLR^WT^; of all the variants, only TLR4^I399^ shared a significant overlap. In TLR4^WT^ and TLR4^I399^, the first EVs overlapped significantly (with a normalized overlap value of 0.634), indicating a similar directional space during the evolution of coordinates. The largest magnitude was represented by the first EV; however, the dominant direction of the first eigenvector was not overlapping in TLR4^GI^ (normalized overlap = 0.22) and TLR^G299^ (normalized overlap = 0.205), indicating a loss of cumulative movements.

The porcupine plots were drawn based on the first EV; the arrows indicate the direction of motion, while the length of arrows indicates the magnitude of EVs. In TLR4^WT^, the two ectodomains were moving away from each other. In TLR4^GI^, the movement of the domains was random. However, in TLR4^G299^ and TLR^I399^, the two domains were moving toward each other as opposed to the movement observed in TLR4^WT^ (Supplementary Figure S10). The dominant motion of TLR4 complexes indicates their propensity of rotation, and if the movements are not correlated, uniform and complementary, this will undermine the probability of forming a complex and will lead to deficiency in subsequent signaling.

### Network and community analysis

Graph theory can accurately predict signaling mechanisms in mutated complexes^31^,^32^. Therefore, we conducted a network analysis to examine the synergism and flow of information in the TLR4/MD2 wild-type and mutated complexes. The residue interaction network was based on the contact map with a threshold of 5 Å derived using StructureViz^33^ and the network was analyzed by RINalyzer^34^ in Cytoscape3.4.0. Every amino acid was treated as a node and the interaction it makes with the other amino acids was represented by edges (Table 1).

**Figure 8:**
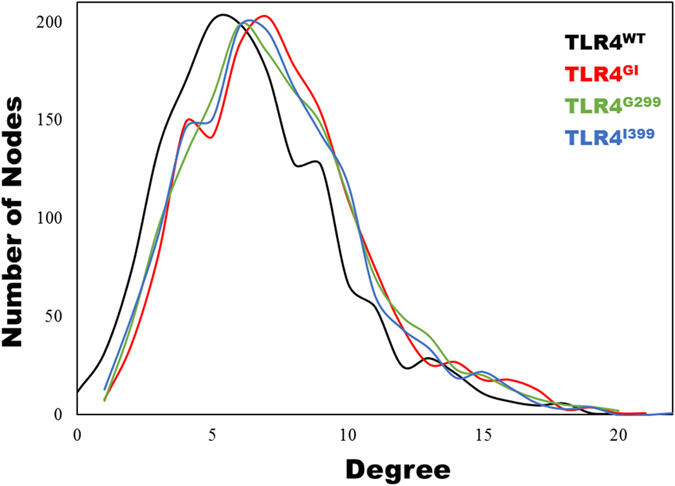
Node degree distribution. The distribution of nodes from various systems. TLR4^WT^ has a peak at 5, whereas the mutated complexes show peaks at approximately 6 or 7.

Analysis of the cluster coefficient (C) and characteristic path length (L) indicated that all the complexes follow small world topology (small values of L and higher values of C). This observation was further supported by the distribution of residue degrees (Fig. 8), which showed bell shaped and Poisson-like distribution curves. The network diameter (~30) and radius (~15) values also indicated a highly regular network symmetry. TLR4 is a clam-shaped molecule, which renders the network density very low. Furthermore, network heterogeneity and average number of neighbors are similar in all the TLR4/MD2 complexes, pointing to considerable network robustness.

The networks were split into communities using the Girvan-Newman algorithm. The community network analysis of TLR4^WT^ showed that the network has 15 communities with 10 isolated communities (Supplementary Figure S11). Similarly, in most of the complexes, the number of communities was similar; 14 communities with 8 isolated communities were found in TLR^GI^, 13 integrated and 8 isolated communities in TLR^G299^, and 14 integrated and 14 isolated communities in TLR^I399^. Because the mutations cannot disrupt the structure of TLR4, the community distribution is preserved.

### Network centrality

To understand the network topology, we examined network centrality, which can highlight the local, as well as global, topological features and reflects the centrality of the most central node in comparison with all the other nodes. For TLR4/MD2 complexes, the betweenness centrality (B_k_) and closeness (C_x_) were calculated per residue (Supplementary Figure S12). The values of B_k_ and C_x_ showed a moderate positive correlation in TLR4^WT^ (0.438, R^2^ = 0.192) and TLR4^GI^ (0.468, R^2^ = 0.214); however, the values showed a weak positive correlation in TLR4^G299^ (0.299, R^2^ = 0.09) and TLR4^I399^ (0.188, R^2^ = 0.035). The distribution of C_x_ was noticeably smooth in all cases because all the complexes were similar.

The central regions of TLR4 and MD2 showed peaks in the B_k_ profile, indicating the density of connections in these regions and their importance in functioning. The notable residues, with a B_k_ value above 0.1, in TLR4^WT^ were: Y292, N365, L385, N433, and S317 from chain A (TLR4); and F345, A366, T457, V338, N433, E336, F358, and R289 from chain B (TLR4). In TLR4^GI^, the notable residues were: N339 from chain A (TLR4); N339, N365, N383, and L385 from chain B (TLR4); and R96 from chain D (MD2). TLR4^G299^ exhibited residues from all the chains, such as E439 from chain A (TLR4), Q436 from chain B (TLR4), R90 and Q92 from chain C (MD2), and R90 from chain D (MD2). The important residues in TLR4^I399^, which displayed B_k_ values above the threshold, were: S317, N365, L385, and F387 from chain A (TLR4), and N365 and F387 from chain B (TLR4).

### Communicating pathways at dimer interface in TLR4/MD2

**Figure 9:**
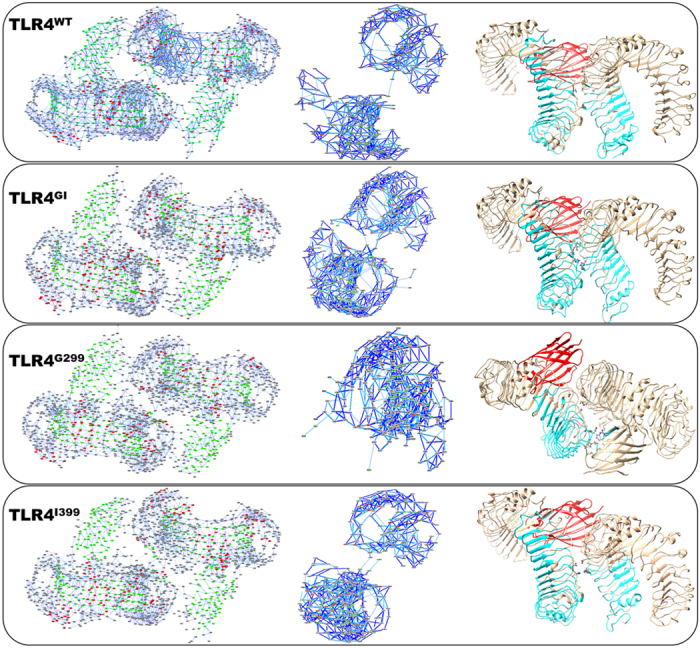
Residue interaction network. The residue interaction network (RIN) was created and analyzed by StructureViz and RINalyzer, respectively, with a non-bonded contact cutoff of 5 Å. In the first panel, RIN representation of every complex is shown; where red indicates helices, green indicates β-sheets, and gray indicates the loop secondary structures. The dark blue line represents main chain interactions, whereas light blue lines represents side chain interactions. The middle panel refers to the pathway starting from position 299 of chain A from TLR4 and highlights the path of communication at dimer interface. Only nodes involved in signal transmission are shown. The last panel shows the structure of the protein onto which the signaling pathway has been mapped. Amino acids involved in signaling are represented by cyan, while the communicating amino acids are represented as a stick model with an atom-based coloring scheme. One MD2 is given in red, while the second, if not involved in signaling, is concealed for clarity.

The network and community analysis of TLR4/MD2 complexes suggested that the presence of allosterism in the TLR4/MD2-LPS complex cooperatively initiates downstream signaling. However, how this allosteric signal perpetuates throughout the complex is still undetermined. For the analysis of this pathway, position 299 was selected as the starting point because the mutation on this site is more influential. Only one pathway in the ectodomain of TLR4^WT^ connects to the counter TLR4 ectodomain. The bridging amino acid in this pathway is N433(A)-N433(B) (where A and B refer to the two domains of TLR4). The full pathway constitutes 328 nodes and 1047 edges in TLR4^WT^ (Fig. 9). However, in TLR4^GI^, the pathway involves two communicating nodes; the first communicating node involves N433 from both chains, while the second node involves H456(A)-H458(B). The total numbers of nodes and edges in this pathway are 424 and 1369, respectively. Higher numbers of nodes and edges weaken the allosteric pathway, resulting in substantially reduced signaling intensity.

In TLR4^G299^, the communicating nodes are also N433(A)-N433(B); however, the signaling pathway could not propagate beyond N433(B) and none of the amino acids was found in the second domain of TLR4. Surprisingly, the TLR4 contains multiple amino acids, such as S438, E439, F463, N464 that communicated with different amino acids of MD2 such as M85, N86, L87, P88, K89 and R90. The numbers of nodes and edges in this pathway were lower at 256 and 604, respectively, as the pathway terminated abnormally. The TLR4^I399^ pathway also showed the two communicating nodes, N433(A)-N433(B) and H458(A)-H458(B); surprisingly this pathway did not propagate and ended at N433. The numbers of nodes and edges in this pathway were 337 and 1088, respectively, which was slightly higher than those in the TLR4^WT^ pathway.

In these pathways, N433 was the key residue that mediated signal transfer between the ectodomains, resulting in reorientation of the TIR domain of TLR4, which perpetuates further signaling^35^. This amino acid may play a key role in the dimerization of TLR4, confirming its importance in the network and signaling communication.

## Discussion

TLR4 is a widely studied cell-surface receptor belonging to the innate immune system. LPS is the canonical ligand that binds TLR4 via MD2, inducing conformational changes that lead to the formation of a complex and downstream signaling. The orientation of the amino acid F126 in MD2 is vital for the initiation of downstream signaling^36^,^37^. There are also SNPs that influence signaling intensity and confer species-specific responses^22^,^38^. The mutations D299G and T399I, in the ectodomain of TLR4, are among those that eliminate signaling activity; however, these residues do not play a role in ligand recognition, nor do they establish contact with MD2. In recent years, crystallographic data have illuminated the structural features of mutated TLR4^39^ and revealing that: (1) overall complex organization is highly conserved, (2) the SNP D299G undergoes local structural changes in LRR10-12, and (3) T399I imparts no structural variation. However, more hydrophobic residues may influence protein folding, cell-surface expression, and stability. Previous reports indicate conflicting results with respect to cell-surface expression and stability of TLR4^14^,^15^. Crystallography studies comprehensively summarize the differences that are induced by these two mutations; however, these differences are minor considering the variable nature of the ectodomain of TLR4 and its participation in ligand recognition^5^,^40^,^41^. This prompted us to examine the dynamic nature of the TLR4 ectodomain with respect to loss-of-function mutations.

MDS calculations revealed that structural features, such as RMSD and Rg, were stable over the entire duration of the MD run (Fig. 1a,b). The number of H-bonds, which are weak interatomic bonds, within and between proteins and LPS, was examined next (Fig. 1c,d). The differential number of H-bonds was consistent over time and can be used to modulate the conformation of a complex in a particular energy basin. Moreover, the vital role of H-bonds in protein structural conformation and interactions is evidenced by the fact that a substitution at one residue (TLR4^G299^) consistently altered the number of H-bonds and resulted in loss of function. An inspection of the Cα RMSF values revealed a coherent and dynamically stable structure of the TLR4^WT^ complex (Fig. 2). However, TLR4^GI^ showed a lower slope value than that of TLR4^WT^, indicating nonlinear behavior and reduced cohesiveness. This indicates its uncorrelated motional behavior, which precludes the complex from exploring other conformational phase space. A SASA analysis revealed that the protein and LPS from TLR4^G299^ had the lowest solvent-exposed area, which may decrease the solubility of the protein and can modulate protein–protein interactions^42^ (Supplementary Figure S2).

Time-dependent structural variation, based on analysis of the secondary structure (Dictionary of Secondary Structure of Proteins classification) over time, shed light on the plasticity of the TLR4 ectodomain. The main variations were observed in TLR4^GI^, in which sequences acquiring secondary structure increased slightly over time. However, TLR4^G299^ and TLR4^I399^ did not exhibit any major structural plasticity in the complex (Fig. 3 and Supplementary Figures S4, S5). Overall, no substantial variation was observed for any complex, likely because the crystal structure showed only a minor deviation at position 299 in the mutated complex. The development of a π-helix was observed in TLR4^G299^ when simulated as a single chain, and briefly in TLR4^I399^ when simulated as a complex; the development of a π-helix was not observed in any other variant. This structural variability may be an artifact; however, it may also indicate the variable structural nature of the TLR4 ectodomain.

The ectodomain of TLR4 forms a partial circle in which one end penetrates the membrane and has the least degree of freedom. However, the N-terminal part can reorient itself to adjust to the second TLR4 as well as to MD2. The reorientation of the atoms in the backbone, from one conformation to another, can be monitored via the internal RCF of the NH bond vector; this is used to calculate the average correlation of the vector in its initial orientation with the orientation after a given period of time^43^,^44^. The RCF data revealed that TLR4^GI^ exhibits a linear decay pattern, whereas TLR4^WT^, TLR4^G299^, and TLR4^I399^ decayed exponentially and achieved a stable value when fitted on a two parameter fit (Fig. 4). Moreover, τc, which provides a good approximation of the molecular weight in a folded protein and can indicate the physical characteristics of the protein, such as aggregation^45^, was 28614.6 ± 4669.3 ps for TLR4^WT^. This value is amidst of TLR^GI^ and single mutant complexes. This may indicate that an appropriate motion, such as that observed in TLR4^WT^, may be necessary for stable complex formation and ultimately for stable formation of a myddosome downstream^46^ (Supplementary Table S1). Because the τc value depends on the molecular weight of the protein, it is surprising that the τc value for TLR4^WT^ (in single and complex form) was comparable in either case however, mutant complexes exhibited twice as compared to their single chain τc values. The τc values for mutant complexes agreed with their molecular weight. However, the τc value for TLR4^WT^ should be greater than the observed value; this disparity may have been caused by the effect of the force field.

The correlative movements differed among the TLR4 variants (Fig. 5), which indicates that the coordination among atoms is hampered by changes in the amino acid sequence and the number of H-bonds present in each complex. Cα atoms are identical in all cases; however, changes in the correlative strength and nature may provide valuable information. Moreover, the distance between dimers increased in TLR4^GI^, and subsequent loss of dimer contacts may likely have hampered signaling efficiency (Fig. 6). TLR4 in endosomes can acquire a tilted conformation to compensate for the effects of pH^47^ that can be influenced by non-covalent bonding. Similarly, in mutants, it is plausible that TLR4 may initiate only TRIF-dependent signaling while the MyD88-dependent signaling is perturbed^18^.

PCA is widely used to investigate the dominant slow movements that influence the overall function in proteins^48^. The two-dimensional graphs between EVs indicate that TLR4^WT^ can be clustered into two separate metastable states that are separated by a substantial energy barrier (Fig. 7). However, this energy barrier may not be sufficient to hinder the change of conformation between the states. Because comparable number of conformations can be observed on either side, it is possible that conformation is reoriented as needed and the same impression could be obtained from dihedral distribution of F126 (Supplementary Figure S6). It may be necessary for a large complex to undergo several conformational changes before attaining a stable and productive conformation. This may not be limited to the state before the formation of an active complex; it can be inferred that TLR4^WT^ can signal from two states. Therefore, it can frequently switch states because both are equally probable during ligand recognition and signaling mediation. The porcupine plots indicated significant alterations in movements of the TLR4 ectodomains, as well as differences in the direction and magnitude of EV (Supplementary Figure S10); however, MD2 was relatively stable in all variants.

Internal communication between distant sites within a protein is fundamental to its function. Internal communication can be investigated using graph theory-based network analysis of protein structures^49^. Such analysis has furnished valuable information for a variety of globular, toroidal, and cylindrical proteins. Unlike MD2, the structure of TLR4 is not globular, but does acquire a cylindrical shape. Therefore, the values of the network diameter and clustering coefficient were similar, as shown in Table 1. The mutations D → G and T → I do not extensively influence structures; therefore, all networks showed similar properties (Fig. 8). Among these, the B_k_ of a node reflects the amount of control this node exerts over the interactions of other nodes in the network^50^, and C_x_ is a measure of how quickly information spreads from a given node to other reachable nodes in the network^51^ (Supplementary Figure S10). The central region of TLR4 and MD2 in all variants showed a higher value of B_k_ and C_x_, indicating their respective roles in signaling and communication. Furthermore, the number of communities within these systems was similar (Supplementary Figure S9), indicating the overall structural stability. Interactions across dimers also showed considerable differences. In TLR4^WT^, N433 is the connecting node between the TLR4 dimers; however, in other systems, other amino acids, in addition to N433, can propagate the signal^19^,^35^. The involvement of two or more nodes from one chain has been shown to weaken the signal^31^ (Fig. 9). Cumulatively, this study has established that a mutation may internally disrupt the signaling pathway, which, in turn disrupts inflammatory signaling.

In most of the analyses, TLR4^G299^ had a distinctly higher impact on loss of function, whereas the structural parameters of TLR4^GI^ could not be differentiated from those of TLR4^WT^. It is possible that the T399I mutation may compensate for the loss of function caused by the D299G mutation. Numerous studies on mutation and correlation report that TLR4^I399^ exerts little to no influence over the functions of TLR4^6^,^52^. Moreover, it is possible that proper TLR4 functioning requires a narrow window of structural plasticity and configuration beyond which the function is compromised. In conclusion, we used MDS to investigate the basis of structural and organizational aspects of TLR4 variants. Because protein function depends on the dynamic nature of the protein, we investigated the dynamic function with respect to loss-of-function mutations and increased differences were observed in the dynamic properties among the variants. Moreover, the ectodomain of TLR4 exhibited different characteristics over time in all variants, and these differences were correlated with abrogated signaling.

## Methods

The crystal structures of wild-type and mutated TLR4 were retrieved from the Protein Data Bank (PDB; accession codes for the WT and mutated forms are 3FXI^24^ and 4G8A^20^, respectively). In order to evaluate the influence of SNPs, four different models were generated: (1) wild-type TLR4 (referred to as TLR4^WT^); (2) a double mutant exhibiting both mutations: G at position 299 and I at 399 (TLR4^GI^); (3) the D299G mutant only (TLR4^G299^); and (4) the T399I mutant only (TLR4^I399^). The respective single mutations were generated using Chimera^53^ by substituting the residue with the most probable rotameric conformation.

### Molecular dynamics simulations (MDS)

The detailed method for MDS is described in our previous publications^22^,^54^. Briefly, GROMACSv5.0.7^55^ was used for simulations. In each simulation, a dodecahedron box filled with TIP3P water model^56^ was generated. For atomic representation, the AMBER99SB-ILDN^57^ force field parameters were selected. The box dimension was properly adjusted to account for minimum image conventions, and periodic boundary conditions were applied in all directions to mimic the infinite system. The Particle mesh Ewald approach was employed for long-range electrostatics^58^ using a 10 Å cutoff distance for both electrostatic and van der Waals interactions, and dispersion correction was applied. Bond lengths were constrained using the LINCS algorithm^59^ that allowed a 2 fs time step in all simulations. Before the simulation, all systems were neutralized, and 100 mM NaCl was added to mimic physiological conditions. Steepest descents and/or conjugate gradient minimization (with a maximum tolerance of 100 kJ/mol/nm) were performed to remove any unfavorable interactions. Each system was prepared for the production simulation by following two-step equilibration. During the first stage, systems were simulated under a constant volume (NVT) ensemble to achieve 300 K by the V-rescale method^60^ for 500 ps. The equilibrated structures from the NVT ensemble were subjected to constant pressure (NPT) equilibration (500 ps) using the Parrinello-Rahman barostat^61^ under an isotropic pressure of 1.0 bar. To avoid configuration changes during equilibration, position restraints were applied to all atoms. Production MDSs were performed for 200 ns in the absence of any restraints. During data collection, the V-rescale thermostat and Parrinello-Rahman barostat were used to maintain the temperature and pressure at 300 K and 1 bar, respectively. Further simulations were conducted with the monomer TLR4 ectodomain for up to 250 ns under similar conditions to evaluate the behavior of the monomeric TLR4 ectodomain.

### Principal components analysis (PCA)

In order to investigate conformational flexibility, the collective motions of TLR4 variants were investigated using PCA^48^. After eliminating the rotational and translational movements, coordinates were superimposed onto a reference structure from which the positional covariance matrix of atomic coordinates and its eigenvectors were calculated. Then, diagonalization was performed on the calculated symmetric matrix by an orthogonal coordinate transformation matrix that yielded the diagonal matrix of eigenvalues. In this diagonal matrix, columns were the eigenvectors corresponding to the direction of motion relative to the initial coordinates. Each eigenvector was associated with an eigenvalue that represented the total mean-square fluctuation of the system along the corresponding eigenvector. The mathematical details have been described previously^62^.

### Dynamics cross correlation and clustering

For the dynamic cross correlation matrix, snapshots recorded every 250 ps, were saved and analyzed using the Bio3D^63^ library as implemented in R. For this analysis, only the Cα was used. To cluster the trajectory, clustering was performed using the Jarvis-Patrick algorithm^64^, which adds a structure to a cluster when this structure and a structure in the cluster, have each other as neighbors and at least three neighbors in common. This clustering method is based on similarity between neighbors, which is calculated by a distance matrix. The number of members can be used to create a cluster for proteins. In this study, this number was set to 10 in deterministic and non-iterative manner.

### Network analyses

To construct the RIN interactively in two-dimensional graphs, we used representative structures from the cluster with the highest number of members from each system. The structure of each protein was modeled as an undirected graph, where amino‐acid residues corresponded to nodes, and contacts between them were represented as edges. The contact criteria of nay residues *i* and *j* was set as the residues are in contact if at least one atom corresponding to residue *i* and any atom from residue *j* were closer than 5.0 Å. This value approximates the upper limit for attractive London–van der Waals forces^65^. An “edge,” which transfers allosteric information from one node to another, is defined as being present between any two nodes provided the absence of a covalent bond, and when the distance between two heavy atoms from the two nodes is less than 5 Å. The strength of the edge between nodes *i* and *j* is defined as the absolute value of the inter-node correlation (*Cij*). The number of connected edges at each node is defined as the degree of the node. Correlation-weighted degree, which is the summation of strengths of all the edges connected to a given node, indicates the importance of the node. After construction of the network, Cytoscape3.4.0, RINalyzer^34^, and NetworkAnalyzer^33^ were used to calculate the topological parameters of the network. The shortest path between any two nodes in the network was identified using the Floyd-Warshall algorithm, and Girvan-Newman algorithm^66^ was employed to create the community based network.

## Additional Information

**How to cite this article**: Anwar, M. A. and Choi, S. Structure-Activity Relationship in TLR4 Mutations: Atomistic Molecular Dynamics Simulations and Residue Interaction Network Analysis. *Sci. Rep.* **7**, 43807; doi: 10.1038/srep43807 (2017).

**Publisher's note:** Springer Nature remains neutral with regard to jurisdictional claims in published maps and institutional affiliations.

## Supplementary Material

Supplementary Information

## Figures and Tables

**Table 1:** Network parameters for wild type and mutated TLR4.

Parameters	TLR4^WT^	TLR4^GI^	TLR4^G299^	TLR4^I399^
Clustering coefficients (C)	0.321	0.319	0.325	0.317
Connected component	4	3	3	2
Network diameter	30	30	29	31
Network radius	15	15	15	16
Network centralization	0.005	0.005	0.005	0.005
Characteristic path length (L)	12.051	12.335	11.827	12.44
Avg.# of neighbors (K)	6.151	6.134	6.156	6.055
No. of nodes	1482	1482	1482	1482
Network density	0.004	0.004	0.004	0.004
Network heterogeneity	0.370	0.367	0.373	0.368
No. of edges	4807	4823	4776	4662
