# Supplementary material for: Structure-Activity Relationship in TLR4 Mutations: Atomistic Molecular Dynamics Simulations and Residue Interaction Network Analysis
Source: Sci Rep. 2017 Mar 8;7:43807. doi: 10.1038/srep43807 (PMC5341570; doi:10.1038/srep43807)
Supplement: Supplementary Information [file srep43807-s1.pdf]

<Supplementary Information>

Structure-Activity Relationship in TLR4 Mutations: Atomistic Molecular Dynamics Simulations and Residue Interaction Network Analysis

Muhammad Ayaz Anwar & Sangdun Choi*

Department of Molecular Science and Technology, Ajou University, Suwon, 443-749, Korea

*Correspondence

Correspondence and requests for materials should be addressed to:

S.C. (sangdunchoi@ajou.ac.kr)

Sangdun Choi

Department of Molecular Science and Technology, Ajou University, Suwon 443-749, Korea

Phone: +82-31-219-2600, Fax: +82-31-219-1615, E-mail: sangdunchoi@ajou.ac.kr

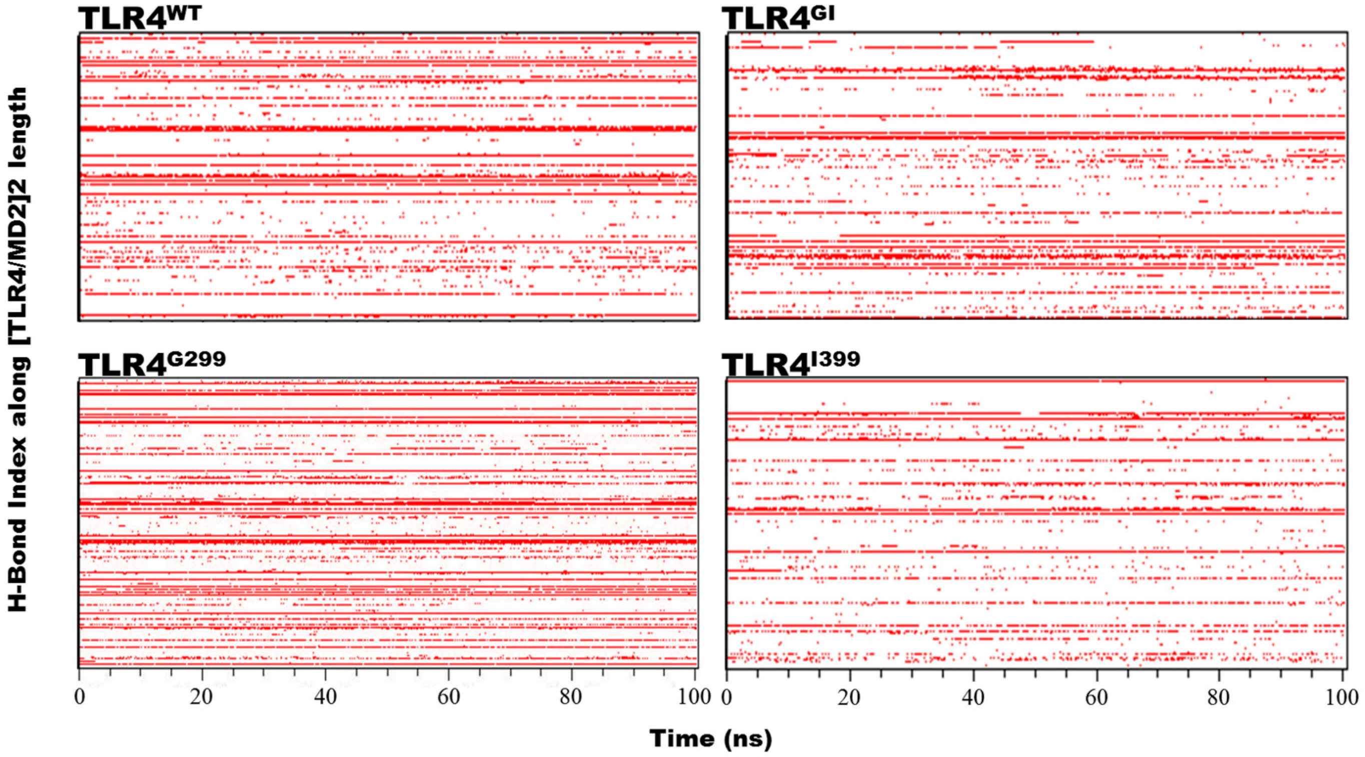

Supplementary Figure S1 | Hydrogen bonding prevalence in TLR4/MD2 complexes. H-bonds have been quantified over the length of TLR4/MD2 complexes for the last 100 ns and plotted along the complex length. Red dots indicate the presence, while white refers to the absence of H-bonds.

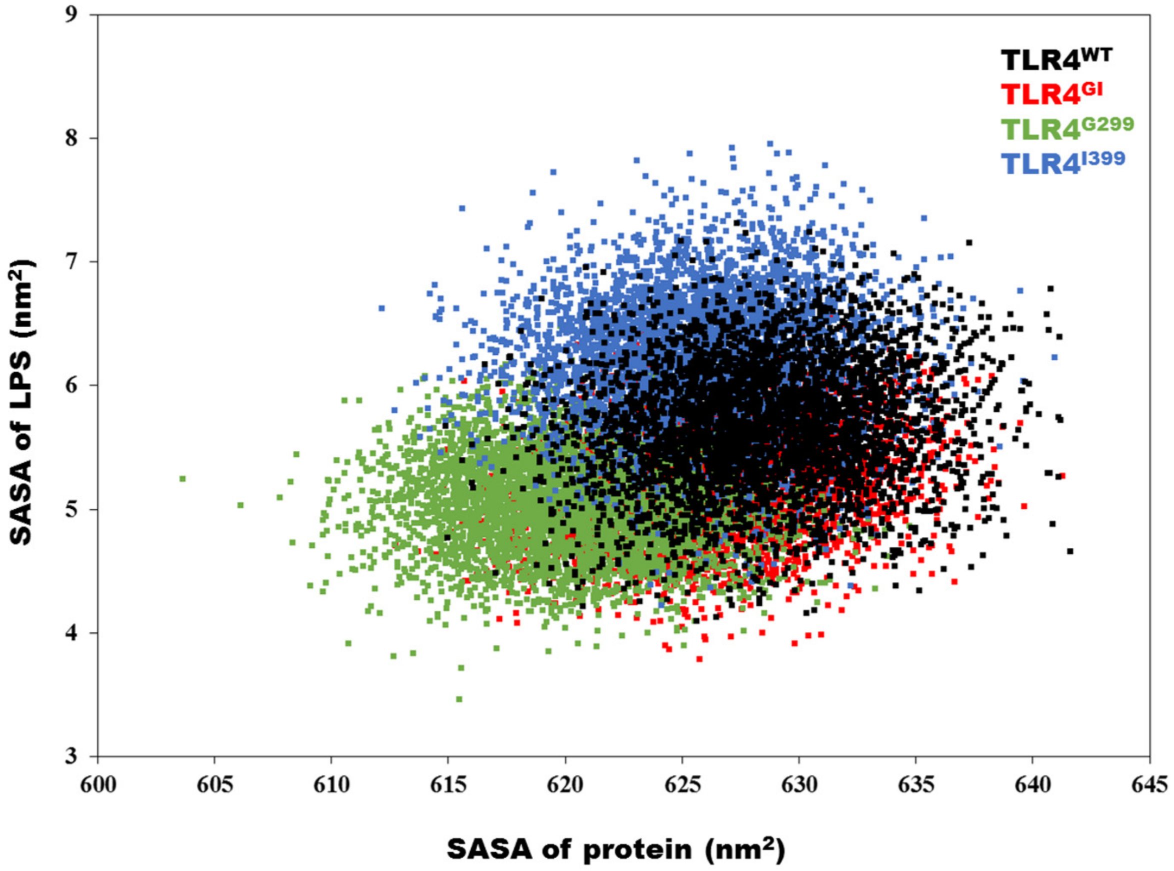

Supplementary Figure S2 | Solvent-accessible surface area (SASA) for TLR4/MD2 and LPS. SASAs were computed for the last 20 ns of the simulation and plotted against each other. The correlation values between the SASA of protein and that of LPS are: $TLR4^{WT}$: 0.12; $TLR4^{GI}$: 0.245; $TLR4^{G299}$: -0.097, and TLR^{I399}: 0.07.

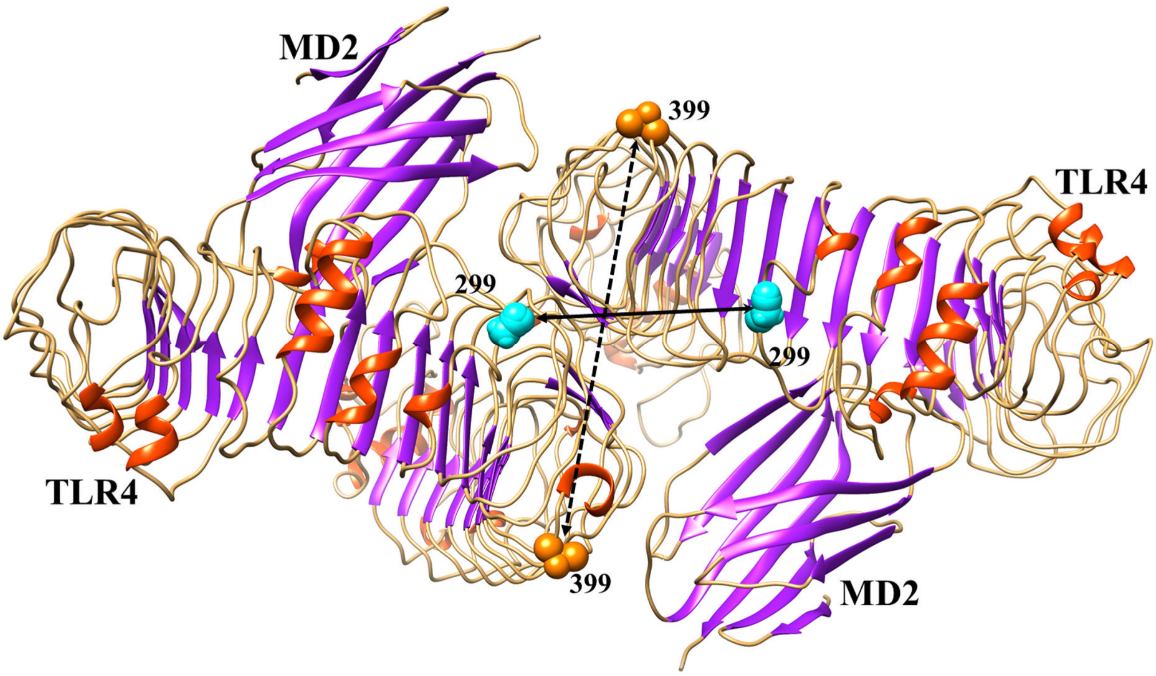

Supplementary Figure S3 | An overall representation of TLR4/MD2 complex showing the distance between position 299 and 399. The complex in cartoon representation where secondary structures have been colored to highlight various secondary structure organization of the complex. Both positions have been represented as sphere, where position 299 is in cyan while position 399 is in orange. This image has been taken from TLR4^{WT}, and LPS has not been displayed for clarity.

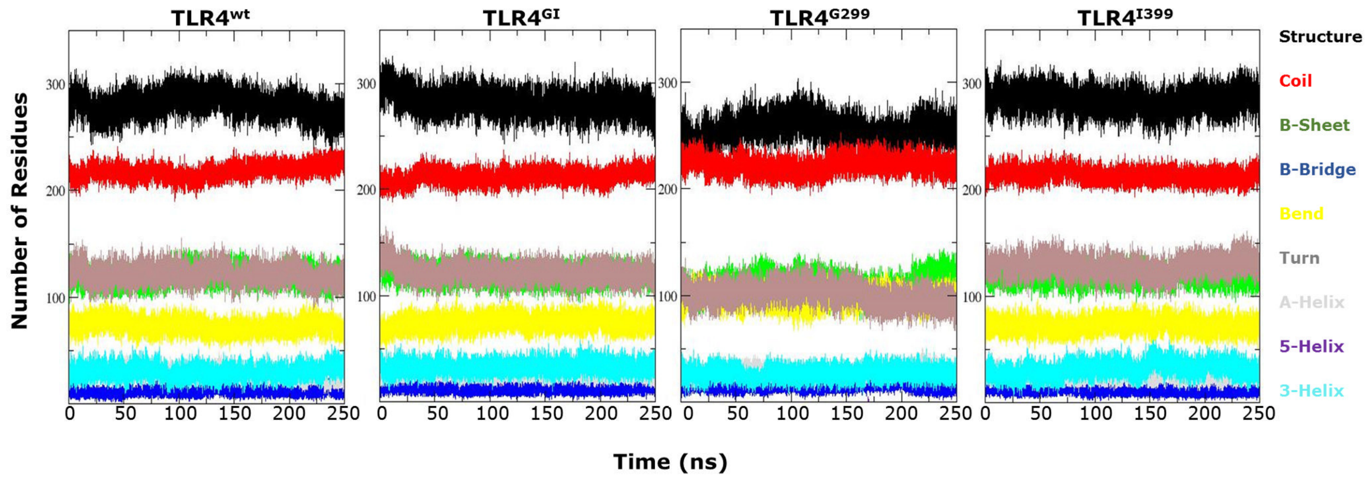

Supplementary Figure S4 | Propensity to form secondary structures during single TLR4 ectodomain simulations. Secondary structures formed by different residues over time are shown. This structural propensity was calculated for the single whole ectodomain of TLR4. The term "structure" includes α-helixes, β-sheets, β-bridges, and β-turns.

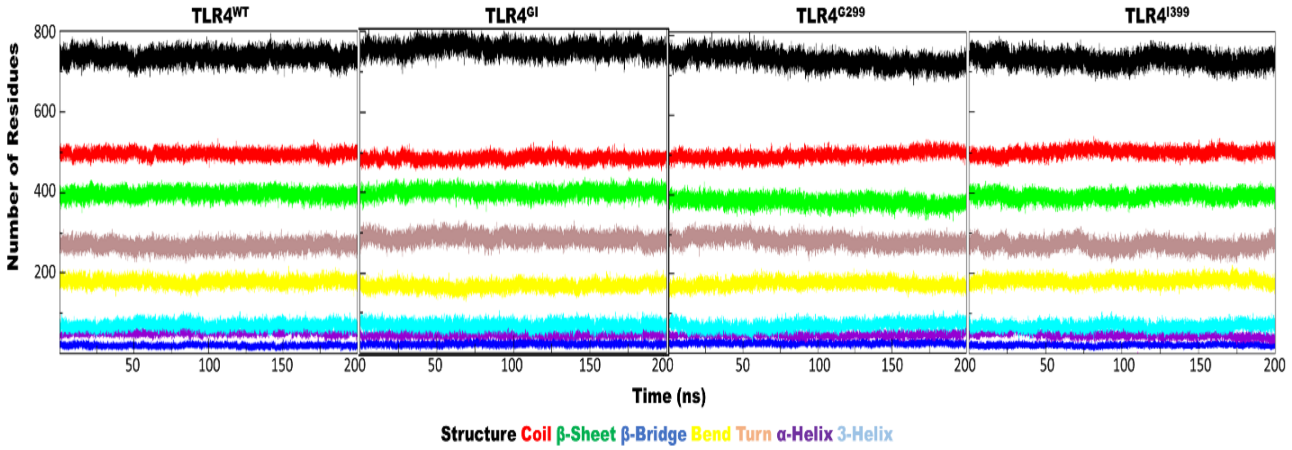

Supplementary Figure S5 | Propensity to form secondary structures during [TLR4/MD2]2 complex simulations. Secondary structures formed by different residues over time are shown. This structural propensity was calculated for the whole complex. The term "structure" includes α-helixes, β-sheets, β-bridges, and β-turns.

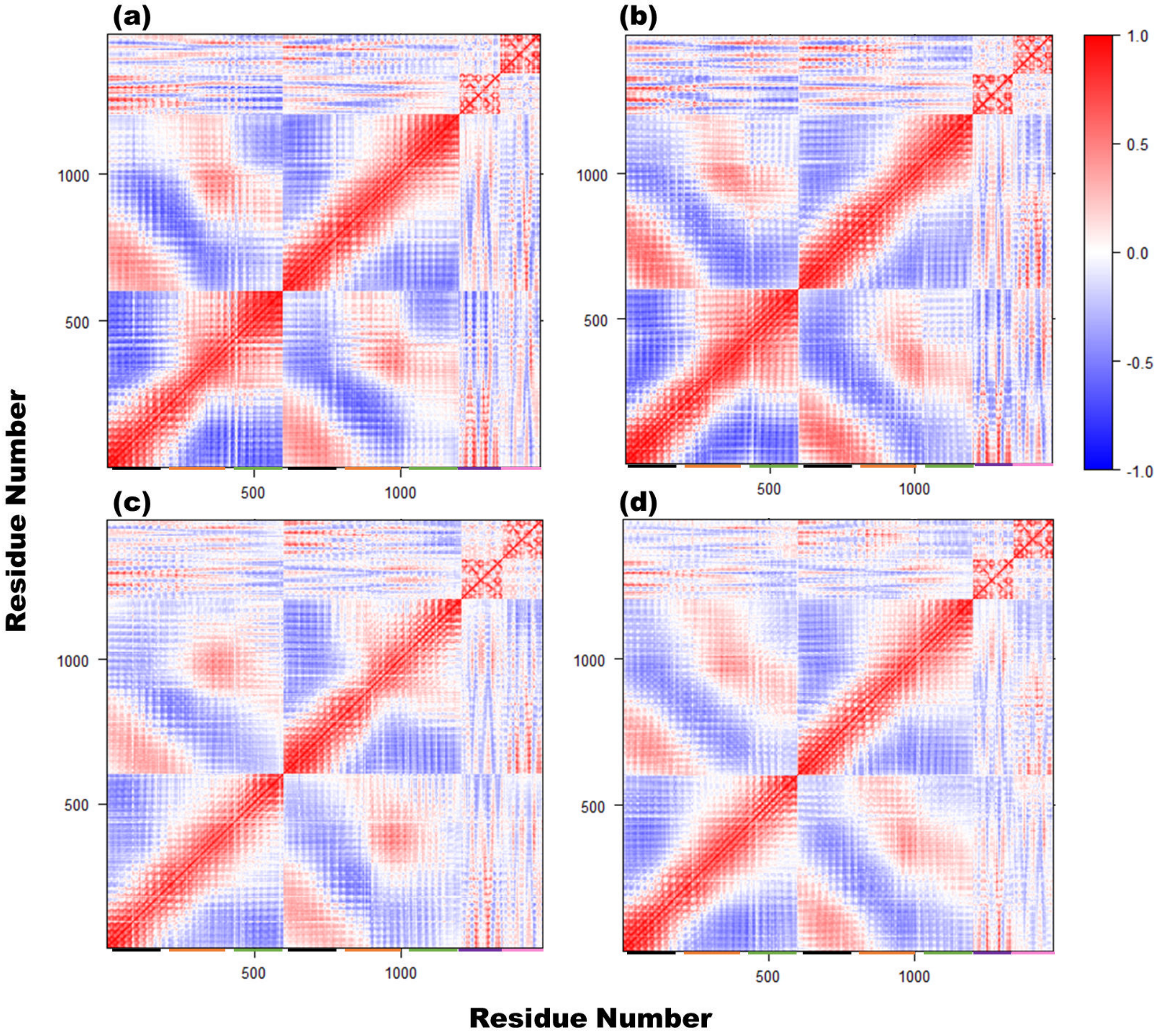

Supplementary Figure S6 | Dynamic cross correlation matrices (DCCM). DCCM from Cα atoms were measured for whole complexes. The diagonal line indicates autocorrelation (100% correlative motion is shown in red); positive correlation between any two atoms is indicated by red and negative correlation is indicated by blue. $TLR4^{WT}$ (a), $TLR4^{GI}$ (b), $TLR4^{G299}$ (c), and $TLR4^{I399}$ (d). The ectodomain of TLR4 is divided into three regions; N-ter region, central region and C-ter region as indicated by black, orange and green bars respectively. Each MD2 has been indicated by magenta and purple bars. The color scale is provided.

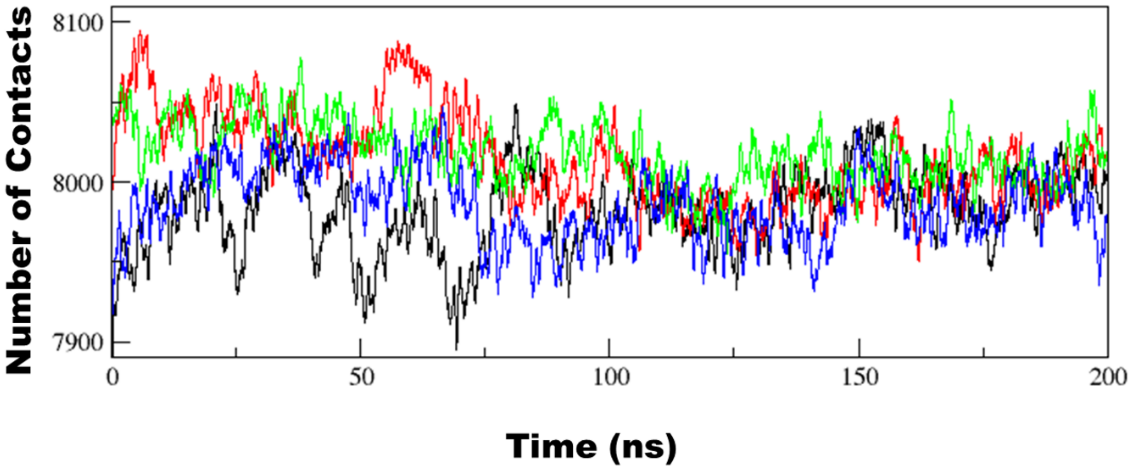

Supplementary Figure S7 | Number of contacts. Contact intensity was calculated between the Cα atom and any other atom within 0.6 nm. The line color represents the complexes; black for $TLR4^{WT}$, red for $TLR4^{GI}$, green for TLR^{G299}, and blue for $TLR4^{I399}$.

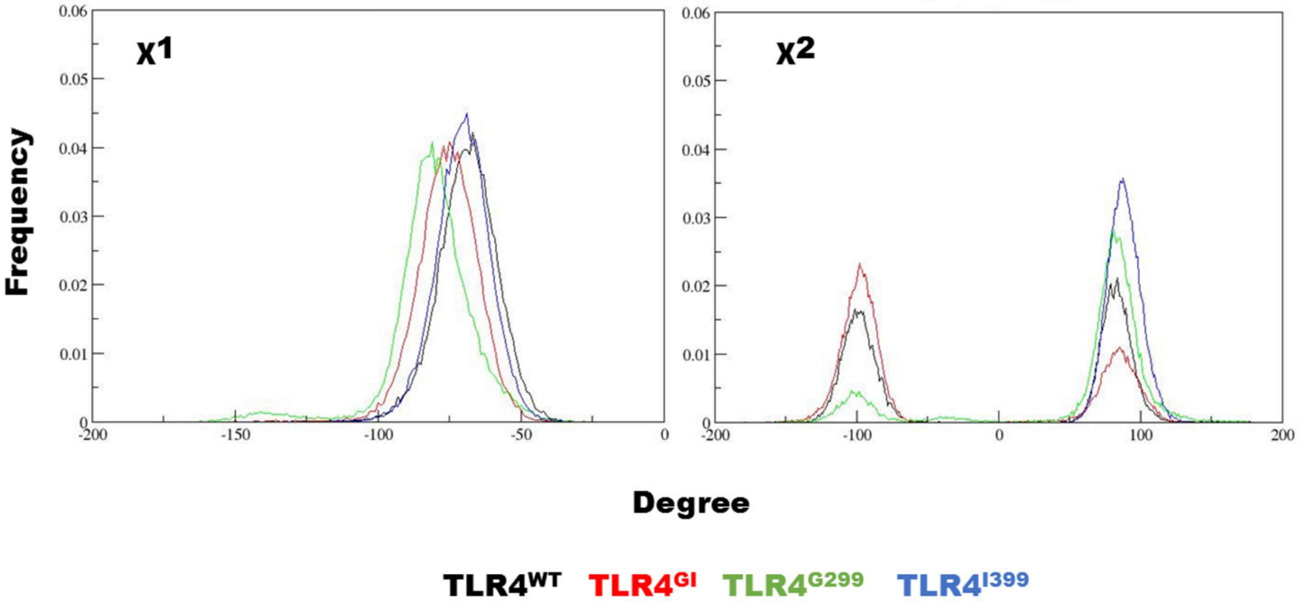

Supplementary Figure S8 | The distribution of χ1 and χ2 of F126 residues from MD2. For χ1, N-CA-CB-CG atoms were considered, whereas for χ2, CA-CB-CG-CD1 were considered.

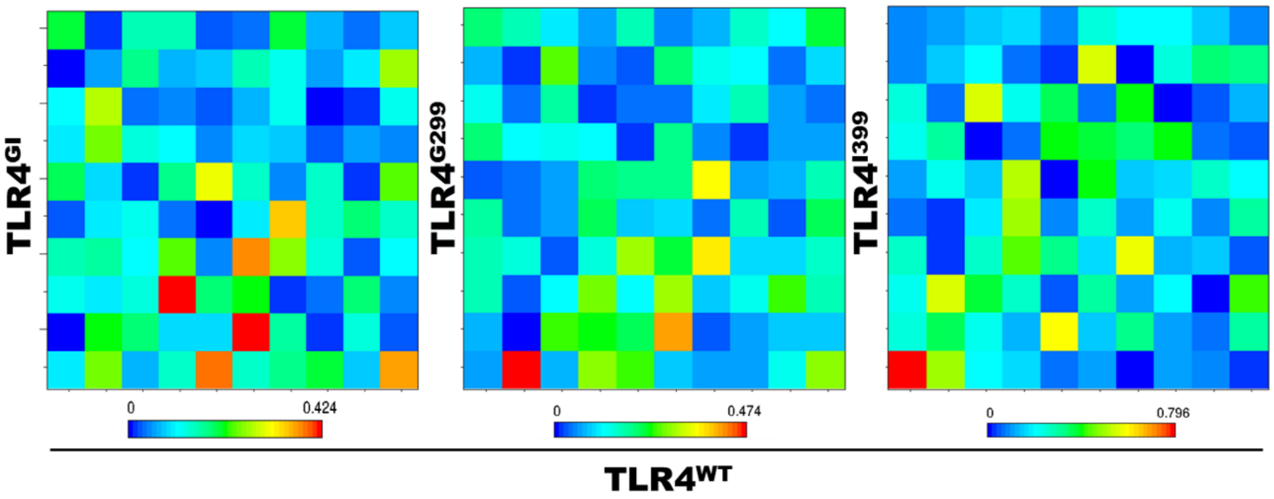

Supplementary Figure S9 | The overlap of covariance matrices. For the overlap, the first 10 eigenvectors were selected because they effectively capture the motions of a protein. Each block represents an overlap of two eigenvectors between any complex and TLR4^{WT}. The color scale is shown at the bottom of each matrix.

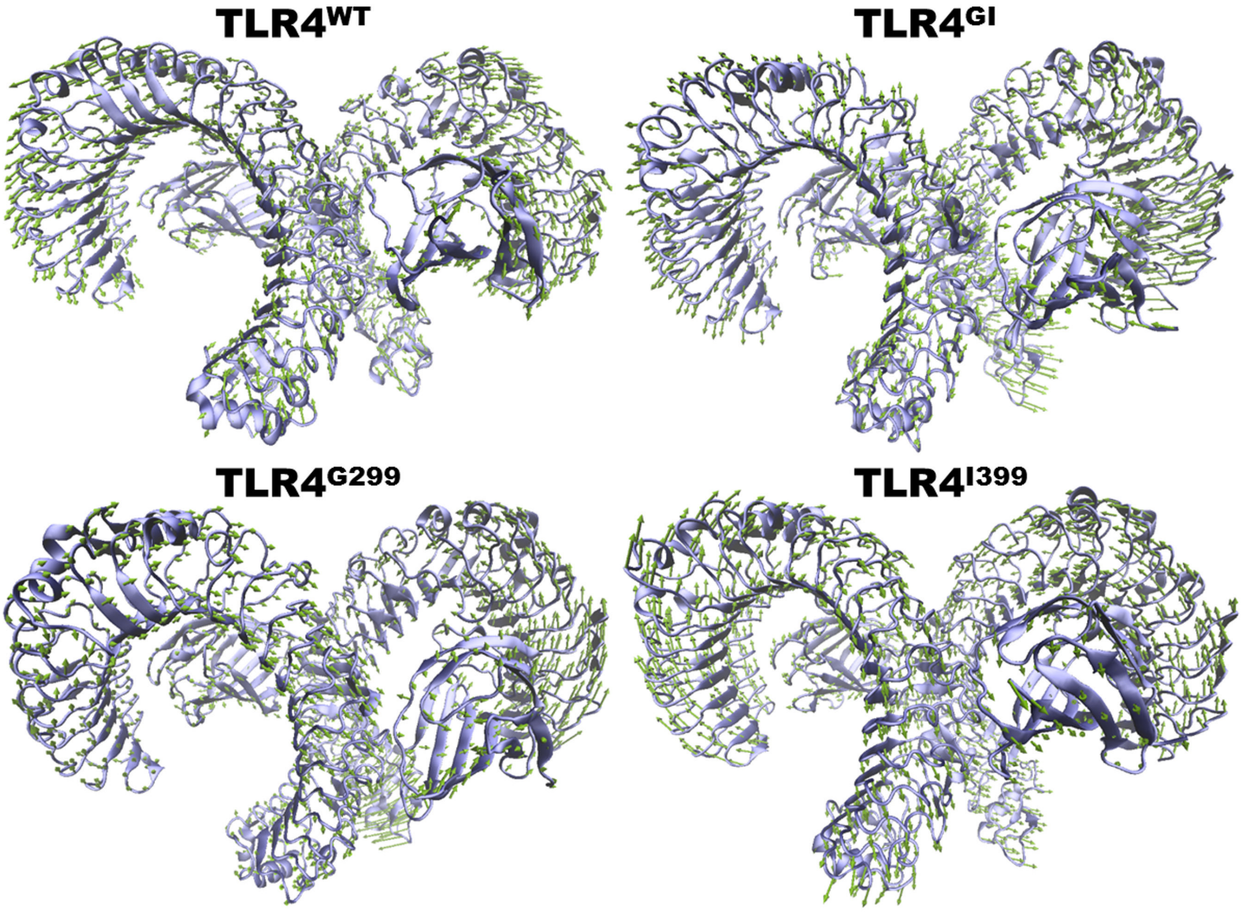

Supplementary Figure S10 | Porcupine plots of TLR4/MD2 complexes. For the porcupine plots, 1200 equidistance conformations were analyzed from the last 100 ns of each MD trajectory to create visual fragmental movements of TLR4 variants. The complexes are represented by carton, while the magnitude and direction of modevectors are shown by green arrows.

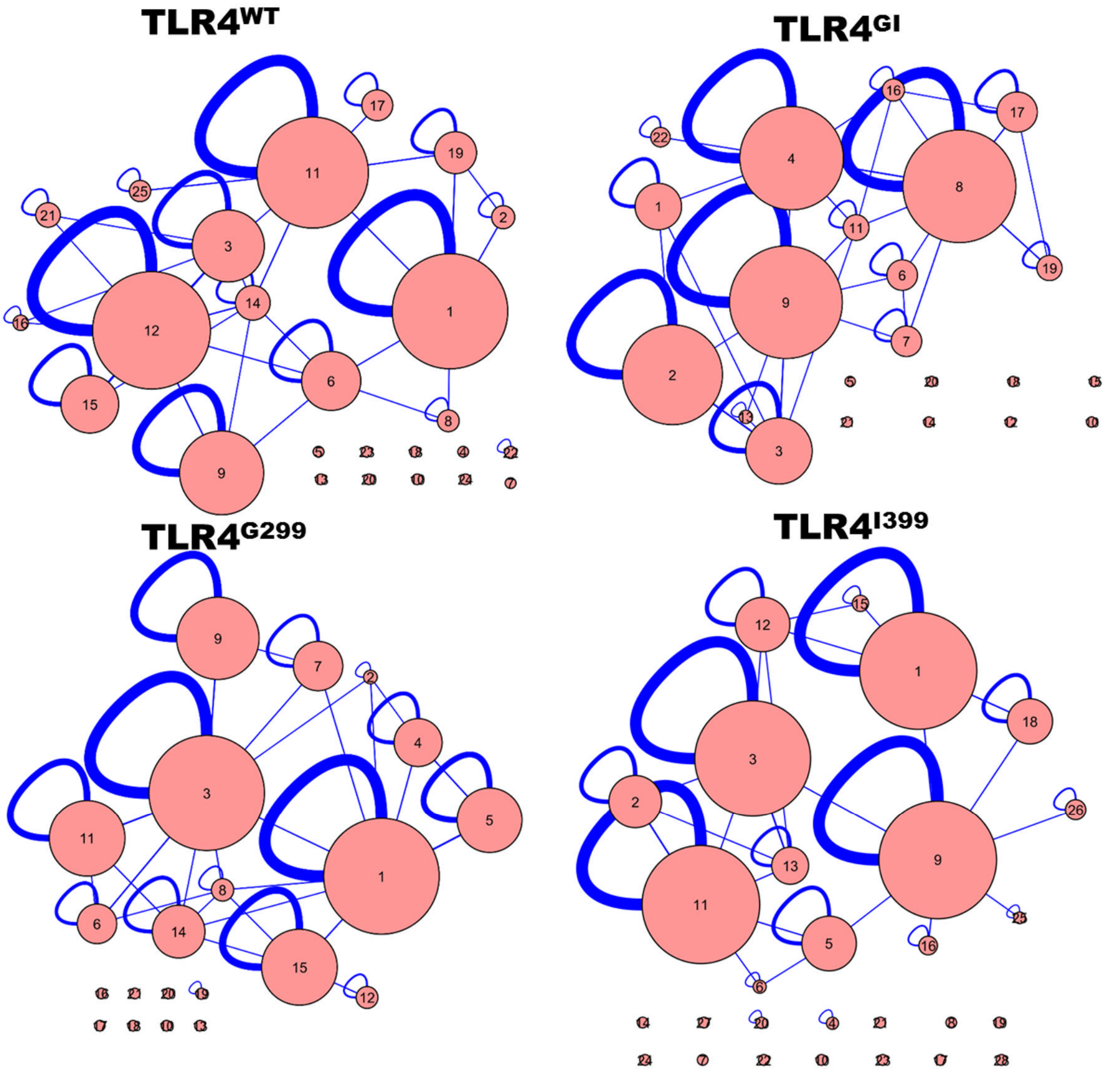

Supplementary Figure S11 | Community analysis of TLR4/MD2. The network of TLR4/MD2 was split into communities based on the Girvan-Newman algorithm. The size of communities is based on the nodes. The isolated communities are provided at the bottom of each variant.

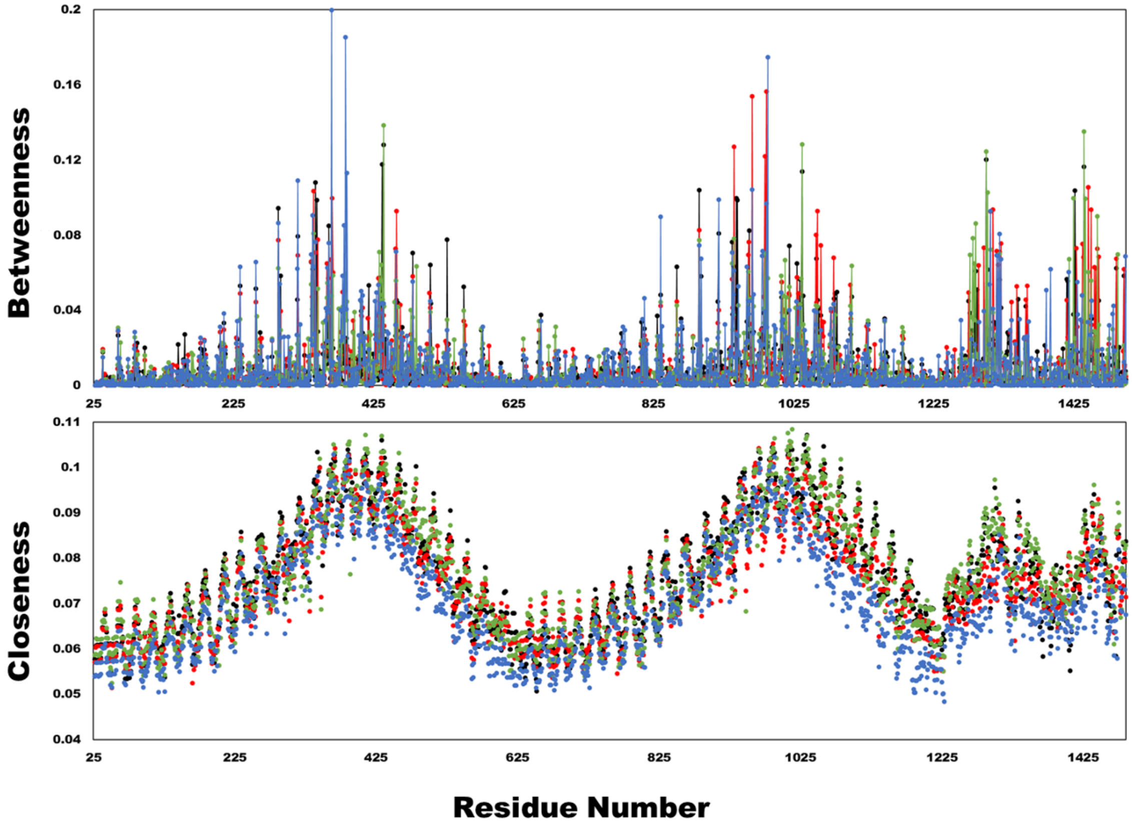

Supplementary Figure S12 | Betweenness centrality (B_k) and closeness (C_x) of TLR4/MD2 complex. The betweenness centrality (B_k) is a measure of the shortest path between each pair of nodes, normalized by the total number of pairs; closeness centrality (C_k) of any node k is the inverse of the average shortest path length.

Supplementary Table S1. Rotational correlation function and its derivative values.

	Rotational correlational time* (ps) ±SD		Isotropic rotational diffusion constant (D_{iso})**	
	**Monomer**	**Hexameric complex**	**Monomer**	**Hexameric complex**
TLR4^{WT}	25268.2±10027.5	28614.6±4669.3	0.65959e-5	0.58245e-5
TLR4^{GI}	13949.8±9588.6	24166.3±6808.9	1.19476e-5	0.68966e-5
TLR4^{G299}	13160.5±8380.2	32772.6±4314.4	1.26642e-5	0.50855e-5
TLR4^{I399}	20496.4±5962.2	34126.8±4050.9	0.81315e-5	0.4883e-5

*Rotational correlation time (τc) is the time required, on average, for a molecule to rotate 1 radian. τc is approximately 1 ns for each 2.6 kDa of protein mass at T = 300 K.

**τc is related to the isotropic rotational diffusion constant: [$\tau c = 1/6D_{iso}$]
